# Modulation of gut microbiota by berberine and metformin during the treatment of high-fat diet-induced obesity in rats

**DOI:** 10.1038/srep14405

**Published:** 2015-09-23

**Authors:** Xu Zhang, Yufeng Zhao, Jia Xu, Zhengsheng Xue, Menghui Zhang, Xiaoyan Pang, Xiaojun Zhang, Liping Zhao

**Affiliations:** 1 State Key Laboratory of Microbial Metabolism, and School of Life Sciences & Biotechnology, Shanghai Jiao Tong University, Shanghai, 200240, China; 2 Shanghai Center for Systems Biomedicine, Shanghai Jiao Tong University, Shanghai, 200240, China

## Abstract

Accumulating evidence suggests that the gut microbiota is an important factor in mediating the development of obesity-related metabolic disorders, including type 2 diabetes. Metformin and berberine, two clinically effective drugs for treating diabetes, have recently been shown to exert their actions through modulating the gut microbiota. In this study, we demonstrated that metformin and berberine similarly shifted the overall structure of the gut microbiota in rats. Both drugs showed reverting effects on the high-fat diet-induced structural changes of gut microbiota. The diversity of gut microbiota was significantly reduced by both berberine- and metformin-treatments. Nearest shrunken centroids analysis identified 134 operational taxonomic units (OTUs) responding to the treatments, which showed close associations with the changes of obese phenotypes. Sixty out of the 134 OTUs were decreased by both drugs, while those belonging to putative short-chain fatty acids (SCFA)-producing bacteria, including *Allobaculum*, *Bacteriodes*, *Blautia*, *Butyricoccus*, and *Phascolarctobacterium*, were markedly increased by both berberine and, to a lesser extent, metformin. Taken together, our findings suggest that berberine and metformin showed similarity in modulating the gut microbiota, including the enrichment of SCFA-producing bacteria and reduction of microbial diversity, which may contribute to their beneficial effects to the host.

The epidemic of obesity-related metabolic diseases, including type 2 diabetes (T2D) and cardiovascular diseases, in both developed and developing countries, have attracted great attentions in the past decades, and thus their etiologies, preventions and therapies have become a matter of global research interest. Human symbiotic gut microbiota, one of the most important internal environment factors^1^, has recently been shown to be closely associated with those obesity-related diseases^2^,^3^,^4^,^5^. Furthermore, evidence from gnotobiotic animal models has revealed the causative role of the gut microbial dysbiosis in obesity^2^,^3^. Recently, we provided the first evidence to show that a single endotoxin-producing strain *Enterobacter cloacae* B29, isolated from the gut of an obese human, caused obesity and insulin resistance in germfree mice^2^. This suggests that the gut microbiota is an important contributing factor in the development of metabolic diseases and may be a potential drug target for such disease treatment. Accordingly, targeting the gut microbiota using dietary interventions^6^, prebiotics or probiotics^7^,^8^ and drugs^9^,^10^,^11^ has been shown to prevent or alleviate the metabolic diseases in the past few years.

Berberine and metformin, two clinically effective drugs for treating T2D and obesity^12^,^13^, have recently been reported to exert their actions through modulating the gut microbiota^9^,^10^,^14^,^15^. As an isoquinoline alkaloid extracted from herbal drugs like *Coptis chinensis* (*Huang-Lian*, a common herb in traditional Chinese medicine), berberine has long been used to treat intestinal infection-related diarrhea in China^16^, and has recently been proven to be clinically effective in treating T2D, dyslipidemia and hypercholesterolemia^13^,^17^. Although many potential mechanisms including the up-regulation of low-density lipoprotein receptor mRNA expression and activation of AMP-activated protein kinase (AMPK) have been proposed^13^,^18^, it is difficult to explain the clinical efficacy of berberine due to its extremely low oral bioavailability^15^,^19^. Recently, Xie *et al.* showed that the antimicrobial activities on both Firmicutes and Bacteroidetes may contribute to the anti-obesity effects of berberine through increasing the intestinal gene expression of fasting-induced adipose factor (*Fiaf*) in mice^14^. We have also previously shown that the berberine-mediated changes of gut microbiota, particularly the increase of short-chain fatty acids (SCFAs)-producing bacteria including *Blautia* and *Allobaculum*, may contribute to the alleviation of inflammation, insulin resistance and obesity by reducing the intestinal endotoxins into the blood^15^. Thus, berberine may have gut microbiota as its primary drug target during the treatment of obesity-related metabolic diseases.

Unlike berberine, metformin has a relatively higher oral bioavailability, and its mechanisms in targeting the host for the treatment of metabolic diseases have been well studied, namely the inhibition of hepatic gluconeogenesis via activation of AMPK^20^,^21^. However, recent studies from several different research groups revealed that the gut microbiota may play an important role in the efficacy of metformin. Shin *et al.* reported that the relative abundance of *Akkermansia* spp. was significantly increased along with the metformin-mediated alleviation of obesity in mice. Introduction of the *Akkermansia* spp. into the gut of diet-induced obese mice improved the host glucose homeostasis^10^, suggesting that the modulation of the gut microbiota may be one of the mechanisms contributing to the antidiabetic effects of metformin. Napolitano *et al.* demonstrated that the human gut microbiome in T2D patients was modified by the treatments of metformin, and these changes were closely associated with the alterations of entero-hepatic recirculation of bile acids and gut hormones^22^. These findings also suggest that the gastrointestinal tract is an important target of metformin.

Taken together, these studies indicate a role for both berberine and metformin in modulating the gut microbiota. However, the direct comparison of these two drugs in modulating the gut microbiota is still lacking, and it is unclear whether they share a common mechanism to induce the shift of gut microbiota shifts. Thus, in this study, we utilized the next generation sequencing (NGS) technique and multivariate statistics to compare the structural changes of gut microbiota induced by berberine and metformin during the treatment of high-fat diet (HFD)-induced obesity in rats. This study provides a direct comparison for the modulations of gut microbiota structure by the treatments of berberine and metformin, which may help to improve our understanding on the host-microbe interactions during the treatment of metabolic diseases and the mechanisms of the two drugs.

## Results

### Berberine and metformin attenuated the high-fat diet-induced obesity

**Figure 1:**
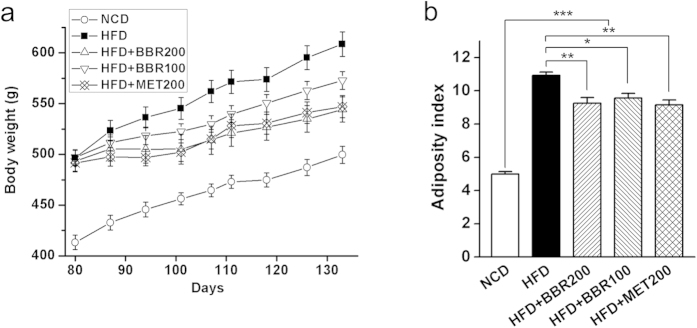
Anti-obesity effects of berberine and metformin in high-fat diet-induced obese rats. (**a**) Body weight; (**b**) Adiposity index, calculated according to the following formula: 100 × (epididymal fat weight + perirenal fat weight)/body weight. Differences were assessed by one-way ANOVA, ^*^P < 0.05, ^**^P < 0.01, ^**^P < 0.001.

Obesity in rats was induced after being fed with high-fat diet (HFD) for 10 weeks (Fig. 1a). Berberine or metformin were then orally administrated for 8 weeks while the rats were continued on HFD. Both berberine (100 mg/kg body weight, HFD + BBR100; 200 mg/kg body weight, HFD + BBR200) and metformin (200 mg/kg body weight, HFD + MET200) treatments obviously attenuated the increase of body weight (Fig. 1a) and inhibited the accumulation of body fat when compared with the HFD group (Fig. 1b). In addition, berberine showed a dose-dependent efficacy in mitigation of the body weight gain, and similar mitigation effects were observed for high-dose berberine and metformin treatments (Fig. 1a). The high dose of berberine significantly reduced the overall food intake during the treatment period, while no significant inhibition was observed for the other groups (Figure S1).

### Response of the gut microbiota structure to berberine and metformin in HFD-fed rats

**Figure 2:**
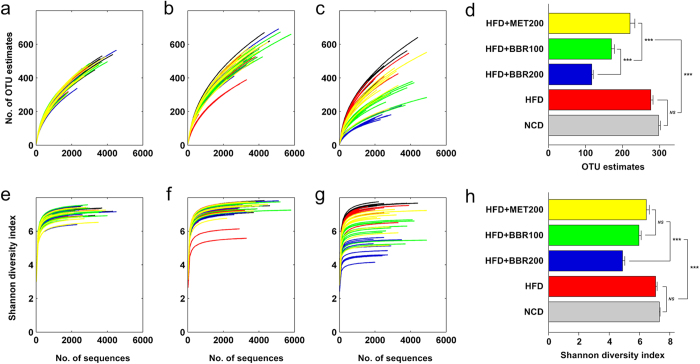
Diversity and richness of the gut microbiota in rats. (**a**–**c**) shows the Rarefaction curves at 0^th^, 8^th^ and 18^th^ week, respectively; (**e**–**g**) shows the Shannon curves at 0^th^, 8^th^ and 18^th^ week, respectively; (**d**,**h**) shows the Rarefaction OTU estimates and Shannon index of each group at the 18^th^ week after rarefying the sequencing depth to 1500 for all the samples. Values are expressed as means ± standard error. Differences were assessed by ANOVA and denoted as follows: ^***^P < 0.001; ^*NS*^ not significant.

High throughput 454 pyrosequencing produced 421,930 high quality sequences from 150 fecal samples collected at week 0, 8 and 18 (with 2,813 ± 1,024 sequences per sample). The high quality sequences were then delineated into 6,933 operational taxonomic units (OTUs) at the similarity cutoff of 98% as per the previously reported method^15^. Shannon and Rarefaction analysis showed that, although new phylotypes can be obtained by additional sequencing, most of the gut microbial diversity in each sample was captured with the current sequencing depth (Fig. 2a–f). After rarefying the sequencing depth among all the samples using bootstrap (1,500 reads per sample), Shannon diversity index and rarefaction OTU estimates were calculated. There was no significant difference in the richness (as indicated by rarefaction OTU estimates) and diversity between HFD and NCD groups in this study, while berberine and, to a lesser extent, metformin, significantly reduced both the richness and diversity of the gut microbiota in rats (Fig. 2g,h).

**Figure 3:**
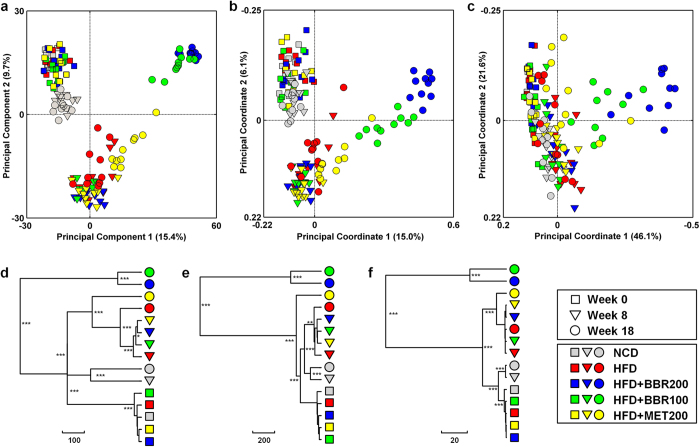
Responses of the structure of the gut microbiota to berberine and metformin during the treatments of obesity in rats. (**a**) PCA score plot; PCoA score plot based on unweighted (**b**) and weighted (**c**) UniFrac metrics. Clustering of the group means based on the Mahalanobis distances calculated using MANOVA based on the PCA (**d**), unweighted UniFrac PCoA (**e**) and weighted UniFrac PCoA (**f**). P values were indicated as: ^*^P < 0.05, ^**^P < 0.01, ^***^P < 0.001.

The overall structural changes of the gut microbiota were then analyzed using unsupervised multivariate statistical methods including principal component analysis (PCA) and UniFrac distance-based principal coordinate analysis (PCoA). PCA scores plot showed that the gut microbiota of NCD group represented a slight structural shift along the second principal component (PC2) with advancing age. The HFD diet feeding resulted in a much greater shift along the same direction (Fig. 3a). The treatment of berberine significantly reverted the HFD-induced variations along PC2 but markedly shifted the structure along PC1 in a dose-dependent manner. Metformin modified the gut microbiota structure with a similar trend to berberine, but to a much lesser extent (Fig. 3a). Both unweighted and weighted UniFrac analysis showed a similar pattern of changes of the gut microbiota in response to berberine and metformin, with a greater distinction in segregation by unweighted UniFrac analysis compared to the weighted one (Fig. 3b,c). Multivariate analysis of variance (MANOVA) based on the PCA (Fig. 3d), unweighted UniFrac (Fig. 3e) or weighted UniFrac (Fig. 3f) analysis also showed great modulating effects of berberine and metformin on the gut microbiota structure. Berberine induced the most significant gut microbial variations which formed a separate cluster in all the three analysis. Metformin treatment resulted in obvious changes of gut microbiota, however, compared to berberine-induced variations, they were much similar to those of HFD groups as revealed by the MANOVA clustering, particularly for PCA- and weighted UniFrac-based analysis (Fig. 3d–f).

### Key phylotypes of gut microbiota modulated by berberine and metformin

**Figure 4:**
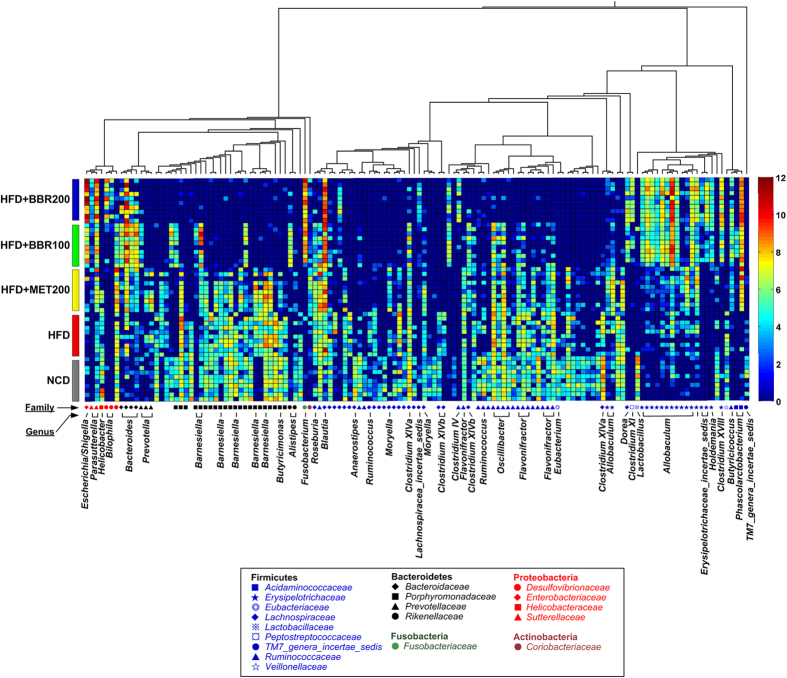
Heatmap of 134 most predictive OTUs. The color of the spot corresponds to the normalized and log-transformed relative abundance of the OTUs. The OTUs are organized according to their phylogenetic positions. The taxonomic assignment of each OTU was determined by RDP classifier and the genus name was labeled in the graph.

Nearest shrunken centroid (NSC) analysis was used for the identification of the gut microbial phylotypes, which were different among the 5 groups at the 18^th^ week. A cross-validated accuracy of 94% was achieved for group prediction based on the NSC model at a threshold of 3.250. A total of 134 most predictive OTUs were identified based on the NSC model (Fig. 4 and Table S1). Among the 134 key OTUs, 85 were decreased or eliminated by both doses of berberine, while 35 were enriched; 70 were decreased by metformin, while 56 were increased. Interestingly, there was a considerable overlap (60 out of 70 OTUs decreased by metformin) between the down-regulated OTUs by berberine and metformin (Table S1). Seven OTUs belonging to *Blautia* (U00003193), *Bacteriodes* (U00000006, U00000261 and U00000076), *Butyricoccus* (U00000374), *Phascolarctobacterium* (U00000235), and *Parasutterella* (U00000494), were significantly increased by both berberine and metformin (Figure S2). The total relative abundance of these 7 OTUs reached a median of 16.2% in HFD + BBR100 group, 22.6% in HFD + BBR200 group, 9.3% in HFD + MET200 group and 1.7% in HFD group (Table S1). Thirteen OTUs belonging to the genus of *Allobaculum* were identified, 9 of which were significantly increased by both doses of berberine while the other 4 were significantly increased by metformin (Fig. 4 and Table S1).

Taxon-based analysis also revealed marked changes of the gut microbial compositions in response to both berberine- and metformin-treatments. A total of 8 in 13 phyla, 17 in 22 classes, 16 in 30 orders, 29 in 51 families, and 63 in 110 genera were significantly different among groups as assessed by the Kruskal-Wallis test (p < 0.05, Table S3). At the phylum level, high-dose berberine significantly decreased the relative abundance of Bacteroidetes, while no obvious change was observed in low-dose berberine and metformin groups. Both berberine and metformin showed no modulating effect on Firmicutes, while Proteobacteria were increased by both treatments. In addition, berberine showed selective enrichment on Fusobacteria, while metformin demonstrated sole effect on Verrucomicrobia. At the genus level, both drugs showed enriching effects on *Allobaculum*, *Bacteroides*, *Blautia*, *Butyricicoccus*, *Lactobacillus*, *Phascolarctobacterium*, *Parasutterella* and *Klebsiella*, and inhibiting effects on *Clostridium* XlVa, *Flavonifractor*, *Lachnospiracea_incertae_sedis*, *Roseburia*, *Clostridium* XI, etc. Metformin’s effect on *Lactobacillus* and *Klebsiella* exceeded that of berberine, while berberine showed greater effects on *Allobaculum*, *Blautia*, *Bacteroides* and *Butyricicoccus* compared to metformin. Some genera including *Dorea*, *Clostridium* XVIII and *Fusobacterium* were only increased by berberine, while *Prevotella* and *Akkermensia* were solely increased by metformin.

**Figure 5:**
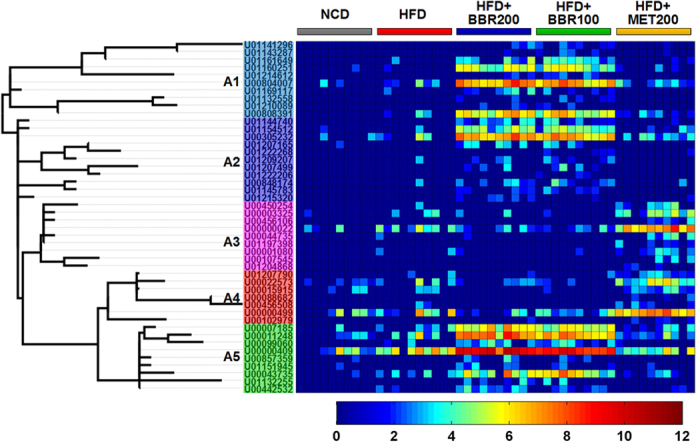
Modulations of *Allobaculum* by berberine and metformin. Each spot of the heatmap corresponds to the normalized and log-transformed relative abundance of the OTU in each sample. The OTUs are organized according to their phylogenetic positions. The home-defined subgroup names (A1, A2, A3, A4 and A5) were labeled according to their phylogenetic positions and distributions among groups.

Among all the 166 observed OTUs belonging to *Allobaculum* in the current dataset, 46 (accounting for 90–100% of the total abundance of this genus in each sample) were significantly different among groups. Interestingly, the 46 OTUs showed an obvious division into 5 phylogenetic subgroups (10 OTUs in subgroup A1, 11 in A2, 9 in A3, 7 in A4, and 9 in A5; Fig. 5), which differently responded to the treatments of berberine and metformin. Subgroup A1, A2 and A5 were enriched by the treatment of berberine, while A3 and A4 were enriched by metformin (Fig. 5 and Table S2), indicating that berberine and metformin showed different modulations on *Allobaculum* at the species level.

### Associations between the gut microbiota composition and obesity phenotypes

To demonstrate whether the variation of gut microbiota structure was associated with the obese phenotypes, we performed an association analysis based on the partial least squares (PLS) regression model using all the 6,933 OTUs obtained in this study. Both adiposity index and body weight were well predicted by the gut microbiome data with a goodness of prediction (Q^2^) value of 0.76 and 0.40, respectively. Significant correlations between the true and predicted body weight (Pearson’s R = 0.64, P < 0.001) and adiposity index (Spearman’s R = 0.60, P < 0.001) were observed (Figure S3). Interestingly, the predicting performance retained using only the identified 134 key OTUs, which achieved Q^2^ values of 0.70 and 0.37, and R value of 0.53 (P < 0.001) and 0.63 (P < 0.001) for predicting the adiposity index and body weight, respectively.

## Discussion

In this study, we found that the fecal microbiota of HFD-fed rats obviously changed during the treatments of berberine and metformin, which is in agreement with previous individual studies for these two drugs^10^,^15^. It is still difficult to distinguish whether those gut microbiota variations were the causes or consequences of the drug treatments. However, the direct comparison of berberine- and metformin-mediated gut microbial changes would help to understand the role of gut microbiota in their efficacies against obesity and diabetes. We found that both berberine and metformin shifted the gut microbiota structure and significantly reduced the microbial diversity in gut of obese rats. Both drugs showed reverting effects on the HFD-induced structural variations. Accordingly, more than half of the identified species-level bacterial phylotypes (134 OTUs) showed similar response to the treatments of the two drugs. More importantly, the identified bacterial phylotypes showed high performance in predicting the obese phenotypes, indicating that the modulations of gut microbiota by berberine and metformin may be relevant to the development and amelioration of HFD-induced metabolic abnormalities.

Among the 134 OTUs, 7 abundant ones were markedly enriched by both berberine and metformin treatments. The total relative abundance of the 7 OTUs was increased to 10–20% in treatment groups from less than 2% in HFD group. Six out of the 7 OTUs belong to the putative SCFA-producing bacteria, including *Blautia*, *Bacteriodes*, *Butyricoccus* and *Phascolarctobacterium*. This suggests an important role of the SCFA-producing bacteria in the efficacies of both berberine and metformin. Turnbaugh *et al.* have reported that the host adiposity was associated with the increase of the intestinal Firmicutes-to-Bacteroidetes (F/B) ratio and enhanced energy harvesting from food, however, a growing number of studies didn’t reproduce these findings. The energy extraction from food has been shown to be a function of food intake, while the contribution of the gut microbiota to energy extraction was very small^23^. We found neither an increase of the F/B ratio after drug treatments nor any correlation between F/B ratio and body weight or adiposity index (Figure S4 and S5). On the contrary, SCFA-producing bacteria have been previously shown to benefit the host through protecting the mucosa from damage induced by pathogens, supplying colonocyte nutrients, mitigating inflammation, etc^24^,^25^. Decrease of such bacteria has been commonly observed in metabolic diseases including T2D^26^, ageing^27^ and even colorectal cancer^28^. Qin *et al.* compared the human gut metagenome of T2D patients with that of healthy adults, revealing that the patients’ gut harbored a decreased level of butyrate-producing bacteria and an increase in the levels of pathogens like *Escherichia coli* and *Desulfovibrio* sp. 3_1_syn3^26^. Effective therapy of T2D, such as by a traditional Chinese herbal formula (*Gegen Qinlian* Decoction, GQD)^11^, has been also reported to recover the abundance of SCFA-producing bacteria. Furthermore, we previously showed that, during the prevention of HFD-induced insulin resistance, berberine markedly enriched the SCFA-producing *Blautia* and *Allobaculum* in the gut of rats and, accordingly, the intestinal SCFAs were increased as well^15^. Thus, the selective modulations of specific gut microbial phylotypes, particularly the enrichment of SCFA-producing bacteria, may participate in the actions of both berberine and metformin in alleviating the host metabolic diseases. Further evidence from clinical study is still needed, particularly, the metagenomics, metatranscriptomics or metaproteomics should be applied for examining the functional changes of human gut microbiome. In addition, the measurement of intestinal SCFA concentrations, by using chromatography techniques^29^, would provide clues for better understanding the clinical effects of berberine and metformin. Moreover, the isolation and culture of potential SCFA-producing bacteria, and inoculation into germfree animals are necessary for underlying the mechanisms of SCFA-producing bacteria in modulating the host metabolic phenotypes.

In addition to the enrichment of SCFA-producing bacteria, berberine and metformin showed high similarity in inhibiting a wide range of intestinal microbes. The antimicrobial activities of berberine in the gut have been previously reported to be related with its anti-obesity effects^14^. Particularly, it was considered to decrease the intestinal endotoxin into the bloodstream and thus decrease the level of low-grade inflammation and eventually prevent the development of metabolic diseases^15^. Lee and Ko also demonstrated that the treatment of metformin resulted in a significant decrease of microbial diversity in HFD-fed mice, but not in NCD-fed mice^30^. These previous findings suggest that both berberine and metformin may inhibit the growth of pathogens which are related to diet-induced metabolic disorders. In this study, we identified 60 OTUs which were decreased by both berberine and metformin, however, due to the limit knowledge for the functions of most mammalian gut microbes, whether those bacteria are pathogens for obesity is still unknown. In addition, whether the inhibition was a consequence of the growth of SCFA-producing bacteria or a direct anti-microbial effects of the drugs also warrants further study. However, the interactions between the resident beneficial gut bacteria (e.g., SCFA-producing bacteria) and opportunistic pathogens (e.g., endotoxin-producing bacteria and sulfate-reducing bacteria) have recently emerged as a crucial factor for intestinal homeostasis^26^, and thus may be as a potential mechanism involved in the beneficial effects of both berberine and metformin.

Although both berberine and metformin increased the SCFA-producing bacteria and reduced the gut microbial diversity, metformin showed obviously lesser extent compared to both doses of berberine. However, its efficacy in reducing obesity was similar to that of high-dose berberine and surpassed the low dose of berberine, suggesting that the structural changes of gut microbiota in berberine- and metformin-treated rats was not solely the consequence of the body weight loss. In addition, we observed drug-specific modulations of gut mcirobiota by berberine and metformin. In agreement with previous studies^10^, *Akkermensia* was increased in metformin-treated rats, but not in berberine-treated ones. Metformin also showed superior effects on some well-known beneficial bacteria, such as *Prevotella* and *Lactobacillus*, which may be responsible for its effects for treating metabolic diseases. *Allobaculum*, another SCFA-producing genus in the gut^15^, has recently been reported to be an important functional phylotypes^15^,^31^,^32^. We found that both berberine and metformin obviously increased the relative abundance of *Allobaculum* in rats, however, different OTUs from this genus showed different responses to berberine- and metformin-treatments, indicating that the modulations of gut microbiota by the two drugs may differentiate in targeting different species even from the same genus.

Metformin has a much higher oral bioavailability compared to berberine. Pharmacokinetic studies revealed that, in rats intragastrically administered with 100 mg/kg of metformin, approximately 4.39% of the drug was not absorbed and excreted intact into feces^33^. Similarly, in humans, 20~30% of the oral dose was reported to be excreted intact into feces after 0.5 to 1.5 g of metformin was taken orally^34^. This part of the orally taken metformin can directly interact with the gut microbiota in the intestine, while majority of the drug enters the circulating system and peripheral tissues to act on the host cells, including the suppression of gluconeogenesis in liver and improvement of insulin sensitivity^20^. However, recent studies showed that, in addition to diabetes and obesity, metformin was also effective in treating multiple metabolic disorders including non-alcoholic fatty liver^35^, polycystic ovary syndrome (PCOS)^36^, cardiovascular disease^37^, aging^9^ and even cancer^38^. It is difficult to explain the mechanisms of a single compound having multiple actions against different diseases, but the recent evidence on the role of gut microbiota in all the aforementioned disorders^39^,^40^ suggests a reasonable explanation for the multiple efficacies of metformin. More interestingly, berberine has also been reported to have similar effects against those diseases^41^. These findings indicate that the modulations of gut microbiota by both berberine and metformin may represent an important avenue for their multi-actions to the host.

We found that both berberine and metformin treatments reduced the food intake of HFD-fed rats. However, whether the reduction of food intake was a direct effects of the drug treatments or a consequence of the microbiota changes is still unclear. Accumulating evidence has shown that the decrease of food intake by calorie restriction modified the gut microbiota^42^,^43^,^44^,^45^. We previously reported that a life-long calorie restriction (CR) architecturally improved the gut microbiota in mice, which markedly extended the mouse lifespan^42^. However, the gut microbiota have also been shown to be a causative factor for regulating the mouse food intake. For example, Vijay-Kumar *et al.* showed that the transfer of microbiota from toll-like receptor 5 (TLR5) deficient mice, which showed increased food consumption and developed hallmark features of metabolic syndrome, into a wild-type germfree mice conferred most of the metabolic phenotypes to the recipients, including hyperphagia^4^. In agreement with previous CR study^42^, we found that *Allobaculum* and *Lactobacillus* were increased after the treatments of berberine and metformin, while the diversity was decreased which was controversial to the previous findings in CR experiments^45^. In addition, the increase of the genera *Blautia* and *Phascolarctobacterium* were only observed in the current treatment study, which indicates that, although the reduction of food intake may contribute in part to the gut microbiota changes or host metabolic improvements, the direct modulations by berberine and metformin may also be an important factor in mediating their efficacies for obesity.

In conclusion, our findings suggest that, in addition to the difference in individually modulating some specific bacterial phylotypes, berberine and metformin showed similarity in shifting the gut microbiota structure during the treatment of HFD-induced obesity in rats. The selective enrichment of the SCFA-producing bacteria and reduction of gut microbial diversity by both drugs may be the shared mechanisms for improving the gastrointestinal health, and eventually mediate their beneficial effects on the host, particularly in metabolic and cardiovascular diseases.

## Methods

### Animal experiments

All the animal experiments were carried out in strict accordance with the Guidelines for Care and Use of Laboratory Animals of Shanghai Laboratory Animal Center (SLAC), Chinese Academy of Sciences, Shanghai, China. The protocol was approved by the Institutional Animal Care and Use Committee of SLAC (No. 2011-007). All efforts were made to minimize animal suffering.

Fifty specific pathogen-free (SPF) Wistar rats (male, 8–10 weeks old) were purchased from SLAC and were included in this study. Both high-fat diet (HFD, containing 60% fat by energy) and normal chow diet (NCD, containing 10% fat by energy) for rats were also provided by SLAC. Berberine chloride and metformin hydrochloride were purchased from Sigma Chemical Co., USA. All the drugs were given to rats by gavage in 0.5% sodium carboxymethylcellulose (NaCMC, Sigma) solution. Non-treatment groups were given the same volume of 0.5% NaCMC to minimize the effects of gavage procedure.

After two weeks’ acclimatization, all the 50 rats were randomly divided into 2 groups: (1) NCD group (n = 10), conventionally raised with NCD diet; (2) HFD group (n = 40), fed with HFD diet. After 10 weeks of feeding, the HFD-fed rats were then randomly divided into 4 more groups: (1) HFD group (n = 10), continually feed with HFD diet (n = 10); (2) HFD + BBR200 group (n = 10), fed with HFD diet and intragastrically administered with 200 mg/kg body weight berberine once daily; (3) HFD + BBR100 group (n = 10), fed with HFD diet and intragastrically administered with 100 mg/kg body weight berberine once daily; (4) HFD + MET200 group (n = 10), fed with HFD diet and intragastrically administered with 200 mg/kg body weight metformin once every day. From the 10^th^ week, all the animals including those in NCD, HFD and treatment groups were intragastrically given drugs or 0.5% NaCMC. Throughout the duration of the trial, the body weight of each rat was monitored twice weekly and stool samples were collected at 0^th^, 8^th^, and 18^th^ weeks. At the end of the trial, after 12 h of food deprivation, fasting body weight was precisely examined and then all the animals were sacrificed by cervical dislocation. Tissues, including perirenal fat, epididymal fat, liver and cecum (both full and empty) were excised, weighed, and frozen in liquid nitrogen immediately for further analysis. Adiposity index was then calculated according to the following formula^46^: (epididymal fat weight + perirenal fat weight) × 100/body weight.

### 454 pyrosequencing of 16S rRNA gene V3 region of gut microbiota

Total genomic DNA of the gut microbiota was extracted from fecal samples by using InviMag® Stool DNA kits (Invitek, Germany) and bead beating methods as reported previously^15^. Then, bacterial 16S rRNA gene V3 region of each sample was amplified with bar-coded primers, ID-P2 (5′-sampleIDtag-ATTACCGCGGCTGCT-3′) and ID-P3 (5′-sampleIDtag-CCTACGGGAGGCAGCAG-3′), with a touchdown PCR approach as described previously. After purification, the amplicons from each of the sample were equally combined and subjected to a sequencing library preparation according to the manufactory’s manual (GS FLX titanium general library preparation kit, Roche Diagnostics Co., USA). Pyrosequencing was performed in Roche 454 GS FLX titanium platform. The raw data and sequencing sample information have been submitted to the NCBI’s Sequence Read Archive (SRA) database under the accession number SRP056927.

### Bioinformatics and Statistics

Quality control of the raw data was performed as described previously^28^. Briefly, each raw reads was considered for the following criteria and those did not meet all the requirements were excluded for further analysis: (1) the sequence should have a perfect match to the barcode in at least one end; (2) the sequence should have a BLAST match to at least one end of the 16S rRNA gene V3 region primers; (3) the length of the trimmed sequence (without barcodes/primers) should be between 100 nt and 300 nt; (4) there should be no more than two undetermined bases. High-quality reads were then aligned against the Greengenes database using the nearest alignment space termination (NAST) algorithm to remove low-quality sequences, including potential chimeric sequences^47^. In this study, we didn’t apply dedicated chimera detection tools because of the relative short V3 region of 16S rRNA gene sequence^48^ and minimal effects of chimera on diversity of microbial community^49^,^50^. The NAST-aligned sequences were utilized for OTU delineation with DOTUR software^51^. At the similarity level of 98%, a total of 6,933 OTUs were obtained and the most abundant sequence of each OTU in all the samples was selected as the representative sequence.

OTU abundance data was then used for the calculation of Shannon diversity index and rarefaction estimates in QIIME^52^. Taxonomical assignments of representative sequences were determined using RDP classifier with a bootstrap cutoff of 50%^53^. Aligned representative sequences were inserted into a pre-established phylogenetic tree of the full-length 16S rRNA gene sequences in ARB to generate a phylogenetic tree, which was then used for both weighted and unweighted UniFrac analysis^54^. Normalized and log-transformed abundance data were used for PCA and PLS regression analysis in MATLAB 2010b (The MathWorks, Inc., USA). The statistical significance of the separation among groups was then assessed by MANOVA using the PCA or PCoA scores. To identify key OTUs which were important for separating different treatment groups, the OTUs with high contributions to the first two PCs in PCA were subjected to NSC analysis with the Predictive Analysis of Microarrays (PAM) package in R software^55^. An amount of shrinking threshold was determined to minimize the overall cross-validated misclassification rate and the OTUs that survived the thresholding were then selected as key variables.

## Additional Information

**How to cite this article**: Zhang, X. *et al.* Modulation of gut microbiota by berberine and metformin during the treatment of high-fat diet-induced obesity in rats. *Sci. Rep.* **5**, 14405; doi: 10.1038/srep14405 (2015).

## Supplementary Material

Supplementary Information

Supplementary Table S1

Supplementary Table S2

Supplementary Table S3
