# Supplementary material for: Modulation of gut microbiota by berberine and metformin during the treatment of high-fat diet-induced obesity in rats
Source: Sci Rep. 2015 Sep 23;5:14405. doi: 10.1038/srep14405 (PMC4585776; doi:10.1038/srep14405)
Supplement: Supplementary Information [file srep14405-s1.pdf]

Modulation of gut microbiota by berberine and metformin during the treatment of high-fat diet-induced obesity in rats

Xu Zhang[1], Yufeng Zhao[2], Jia Xu[1], Zhengsheng Xue[1], Menghui Zhang[1], Xiaoyan Pang[1], Xiaojun Zhang[1], and Liping Zhao[1,2 *]

[1] State Key Laboratory of Microbial Metabolism, and School of Life Sciences & Biotechnology, Shanghai Jiao Tong University, Shanghai, 200240, China;

[2] Shanghai Center for Systems Biomedicine, Shanghai Jiao Tong University, Shanghai, 200240, China.

[*] Corresponding author, Liping Zhao, Shanghai Jiao Tong University, Shanghai, China; Tel: 86-21-34204877; Fax: 86-21-34204878; E-mail: lpzhao@sjtu.edu.cn or lpzhao3517@126.com.

Supplementary Tables

Table S1 Relative abundance and taxonomy of the 134 key OTUs identified by nearest shrunken centroids analysis.

Table S2 Relative abundance of the 46 significant OTUs from the genus of *Allobaculum*.

Table S3 Taxon-based comparisons of the gut microbiota structure among treatment groups.

Supplementary Figure Legends

Figure S1 Effects of berberine and metformin on the overall food intake of HFD-fed rats. The overall food intakes for each group (n=3) were calculated as the sum during the treatment period. Values are expressed as means ± standard error. Differences were assessed by ANOVA and denoted as follows: * $P<0.05$; ** $P<0.01$.

Figure S2 Relative abundance of highly enriched OTUs by both berberine and metformin. Values are expressed as means ± standard error. Differences were assessed by ANOVA and denoted as follows: * $P<0.05$; ** $P<0.01$.

Figure S3 Scatter plots of the true and predicted body weight and adiposity index. PLS regression was used for the prediction of body weight (a, b) and adiposity index (c, d) with either the whole microbiota structure (a, c) or the identified 134 key OTUs (b, d). The Pearson's coefficient R or Spearman's correlation R was indicated.

Figure S4 The abundance ratio of Firmicutes-to-Bacteroidetes in the gut of rats. The median (central thick lines), 25% and 75% quartile ranges (box width), and upper and lower limits (error bar) of each group are shown in the box plot. Data was also shown as squares besides the box plot. Differences were assessed by the Mann-Whitney test, * $P<0.05$, ** $P<0.01$.

Figure S5 Scatter plot of fecal Firmicutes-to-Bacteroidetes ratio and bodyweight (a) and adiposity index (b). Spearman's correlation coefficient R and P values are indicated.

Figure S1

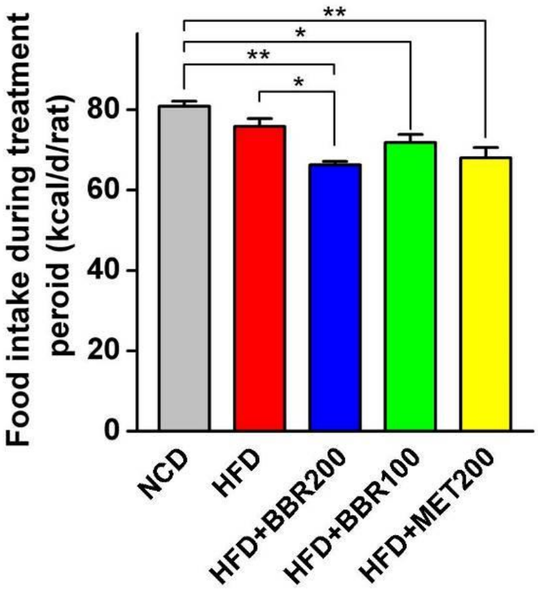
Food intake during treatment peroid (kcal/d/rat)
**
*
**
*
80
60
40
20
0
NCD
HFD
HFD+BBR200
HFD+BBR100
HFD+MET200

Figure S2

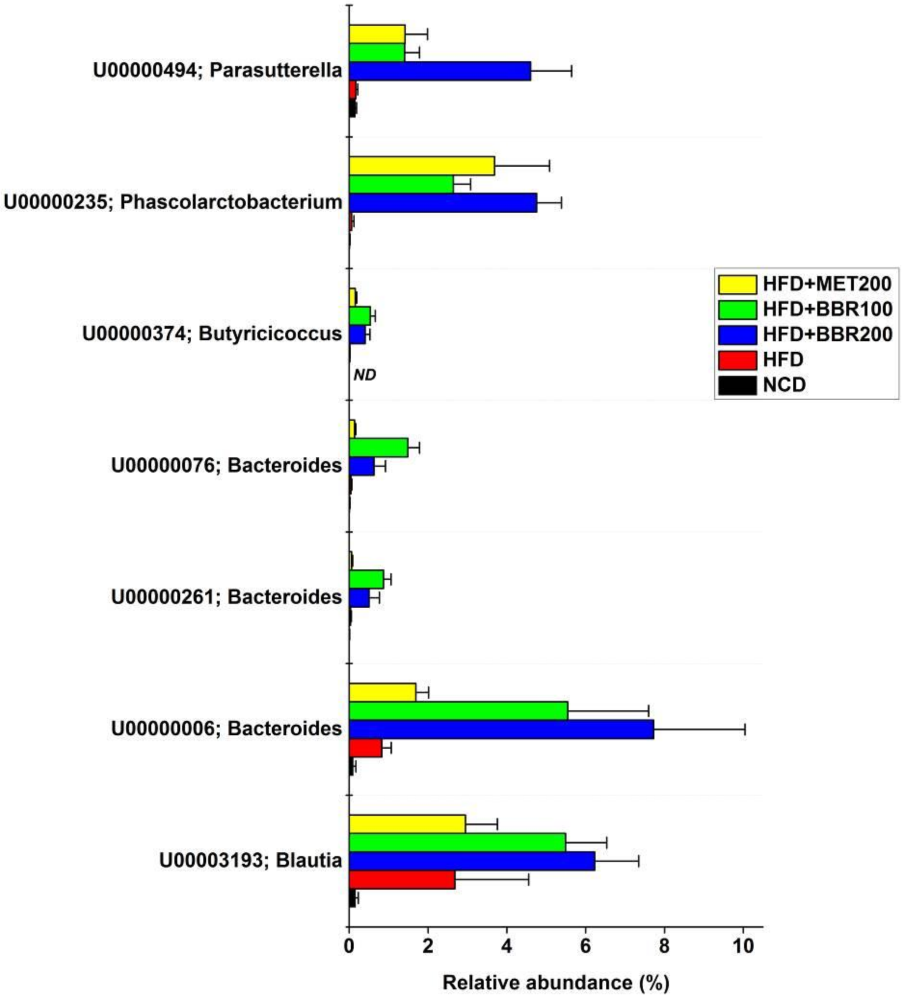

U00000494; Parasutterella
U00000235; Phascolarctobacterium
U00000374; Butyricicoccus
ND
U00000076; Bacteroides
U00000261; Bacteroides
U00000006; Bacteroides
U00003193; Blautia
HFD+MET200
HFD+BBR100
HFD+BBR200
HFD
NCD
0
2
4
6
8
10
Relative abundance (%)

Figure S3

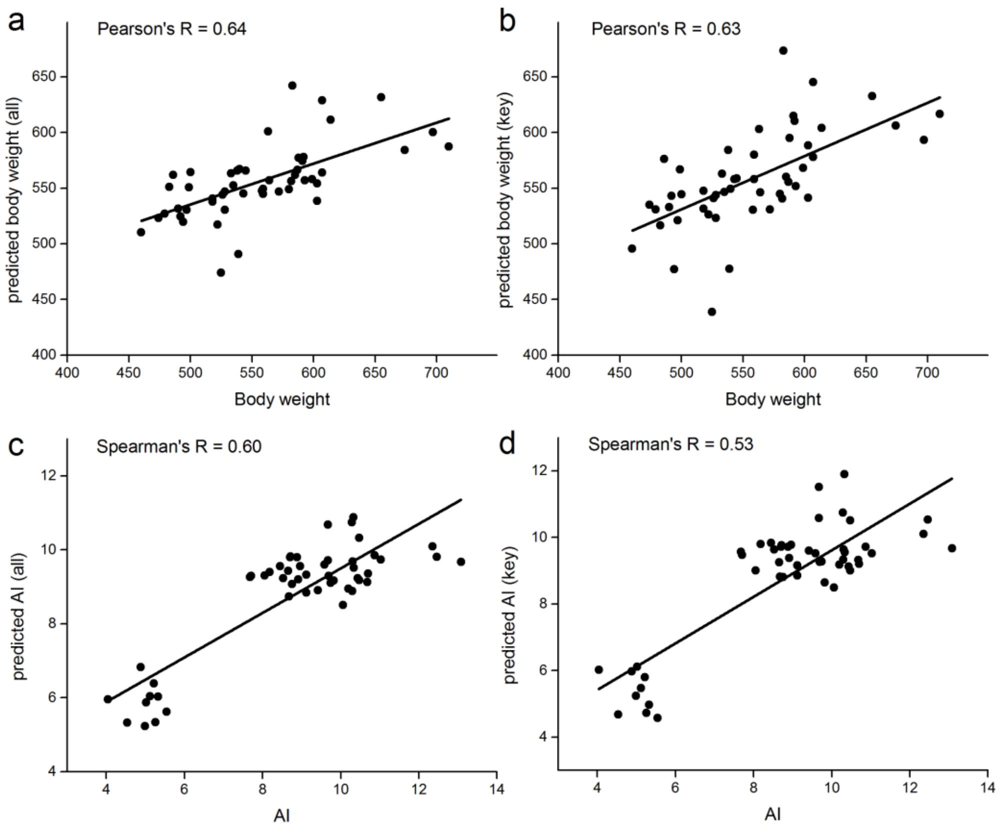
a
Pearson's R = 0.64
predicted body weight (all)
Body weight
b
Pearson's R = 0.63
predicted body weight (key)
Body weight
c
Spearman's R = 0.60
predicted AI (all)
AI
d
Spearman's R = 0.53
predicted AI (key)
AI

Figure S4

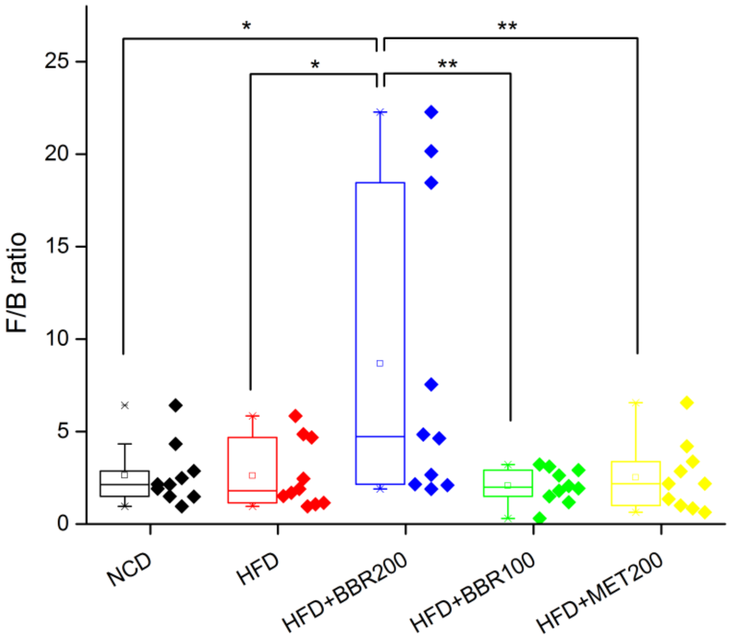
*
**
*
**
25
20
15
10
5
0
F/B ratio
NCD
HFD
HFD+BBR200
HFD+BBR100
HFD+MET200

Figure S5

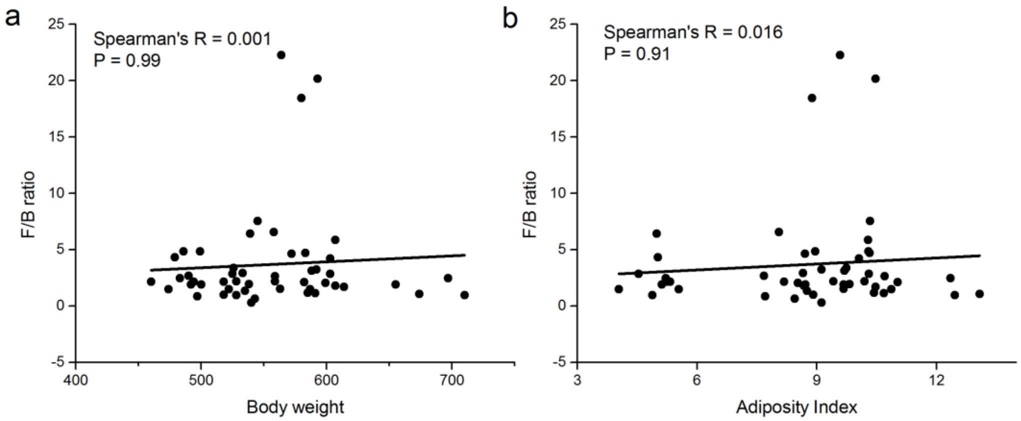
a
Spearman's R = 0.001
P = 0.99
F/B ratio
Body weight
b
Spearman's R = 0.016
P = 0.91
F/B ratio
Adiposity Index
